# Does Type 2 Diabetes Increase the Risk of Hepatocellular Carcinoma in Nonalcoholic Fatty Liver Disease Patients? A Systematic Review

**DOI:** 10.7759/cureus.36079

**Published:** 2023-03-13

**Authors:** Sathish Venugopal, Ravneet k Dhanoa, Tharun Yadhav Selvamani, Shoukrie I Shoukrie, Anam Zahra, Jyothirmai Malla, Ramaneshwar Selvaraj, Ranim K Hamouda, Lubna Mohammed

**Affiliations:** 1 Neurology, California Institute of Behavioral Neurosciences & Psychology, Fairfield, USA; 2 Internal Medicine, California Institute of Behavioral Neurosciences & Psychology, Fairfield, USA; 3 General Surgery, California Institute of Behavioral Neurosciences & Psychology, Fairfield, USA; 4 Orthopaedics and Traumatology, California Institute of Behavioral Neurosciences & Psychology, Fairfield, USA; 5 Surgery, California Institute of Behavioral Neurosciences & Psychology, Fairfield, USA; 6 Internal Medicine/Family Medicine, California Institute of Behavioral Neurosciences & Psychology, Fairfield, USA

**Keywords:** hepatocellular carcinoma (hcc), insulin resistance, diabetes mellitus type 2, diabetes, non alcoholic fatty liver disease

## Abstract

Diabetes is associated with different types of cancers of which hepatocellular carcinoma (HCC) is one among them. In a study comparing patients with diabetes to those who do not have diabetes, it was evident that the risk of HCC is found to increase two-fold in diabetic than that in non-diabetic patients. It is clear that carcinogenesis is advanced due to diabetes in the liver by a variety of mechanisms. We searched PubMed and Google Scholar for articles from 2010 to 2021 that have an association between diabetes, nonalcoholic fatty liver disease (NAFLD), and HCC. For the development of HCC, diabetes is likely related at both the molecular and epidemiological levels. Both diabetes mellitus and hepatic malignancy have the worst impact on mankind socioeconomically. There is a significant relationship between diabetes and HCC independent of alcohol consumption and viral hepatitis. It is noteworthy that not only the elderly but also people of all age groups should monitor their hemoglobin A1C levels. Diet restriction and lifestyle modification can reduce the risk of complications like HCC; the increased physical activity itself can have a major influence on health and can manage comorbidities like diabetes, NAFLD, and HCC.

## Introduction and background

Diabetes is highly associated with various types of cancers including hepatocellular carcinoma (HCC) [1]. In a study of patients with diabetes vs patients without diabetes, the risk of HCC is found to increase two-fold in patients with diabetes more than in patients who do not have diabetes [2]. Carcinogenesis is promoted by diabetes in the liver by a variety of mechanisms such as inflammatory cascades by manufacturing proinflammatory cytokines and reactive oxygen species (ROS) that can cause genetic instability, increase cellular proliferation, and prevent apoptosis in liver hepatocytes [1]. However, it is not possible to explain the occurrence of liver pathologies, cardiovascular events, malignancy, and type 2 diabetes mellitus in histopathologically confirmed nonalcoholic fatty liver disease (NAFLD) patients [3]. Many risk determinants of HCC have already been discussed such as the etiology of cirrhosis, age, gender, race, body mass index (BMI), diabetes, and hepatitis C virus (HCV) genotype [4]. The occurrence of HCC in diabetes mellitus is most likely related at both molecular and epidemiological levels [5]. A recent study evidenced that diabetes is associated with a three-fold higher risk for HCC in NAFLD [1]. NAFLD has raised concern worldwide as an important healthcare problem. It arises in individuals whose liver weighs more than 5 to 10% as a result of excess fat formation [6]. In NAFLD patients, the risk of liver disease progression and development of liver cirrhosis is increased by diabetes [1]. NAFLD-related HCC has been found to increase as a result of the worldwide increase in obesity. NAFLD-related HCC arises more in elderly individuals than HCC related to other age groups [2]. It has been found that patients who have been diagnosed with NAFLD were at higher risk for HCC (HR 6.55 P= 0.001) [3]. NAFLD has been placed as a significant cause of HCC entirely based on risk factors, such as diabetes and obesity, without considering other chronic liver diseases [1].

Diabetes mellitus and liver cancer have the worst effects on mankind socioeconomically [7]. There is a noteworthy relationship between diabetes and HCC independent of alcohol consumption and viral hepatitis [8]. HCC accounts for about 70 to 90% of primary liver cancer, and it is the fifth leading cause of cancer in Europe. The epidemiological data of Europe shows that at least 1 to 13 new cases of HCC account for 1-10 deaths per 100,000 person-years [2]. Geriatric patients who have a low level of serum albumin should be carefully monitored for HCC [1]. Elderly people have been correlated with HCC incidence which shows the importance of comorbidity management because of the greater incidence of comorbidities, such as diabetes mellitus and hypertension, in the older population [5]. In 2018, neoplasm in the liver is found as the sixth most frequent and prevailing cause of cancer-related deaths globally with approximately 841,000 new cases and 782,000 deaths in which HCC is the most common [5]. In this study, we aim to further analyze how diabetes increases the risk of HCC in NAFLD patients.

## Review

Methods

This systematic review followed the Preferred Reporting Items for Systematic Reviews and Meta-Analyses (PRISMA) guidelines [8]. We searched PubMed for articles from 2010 to 2021 that assess the association between diabetes, NAFLD, and HCC, and an additional database, such as Google Scholar, was also included. We included studies in systematic reviews and meta-analyses, cohort studies, case series, and literature reviews. The articles were taken in a way that provides direct or indirect data on the research. We collected data on “complications,” “pathology,” and “pathophysiology” of diabetes, NAFLD, and HCC using the general keywords “Nonalcoholic fatty liver disease,” “Diabetes,” “Diabetes mellitus type 2,” and “Hepatocellular carcinoma" and Medical Subject Headings (MeSH) to build the search strategy using Boolean operators like AND/OR. We included some articles from Google Scholar as an additional database. The population, intervention, comparison, outcomes, and study criteria are important for our eligibility criteria. Table 1 describes the keywords that were used in databases like PubMed, Google Scholar, and search results.

**Table 1:** Keywords and search results

Keyword	Databases	Filters applied	Search results
Diabetes and nonalcoholic fatty liver disease	PubMed	2010–2021 and free full text	3527
Diabetes and hepatocellular carcinoma	PubMed	2010–2021 and free full text	1413
Hepatocellular carcinoma and nonalcoholic fatty liver disease	PubMed	2010–2021 and Free full text	1804
Diabetes and nonalcoholic fatty liver disease and hepatocellular carcinoma	Google Scholar	2010–2021	16,800

Inclusion/exclusion criteria

We took articles that were published from 2010 to 2021 and included only articles that are written in the English language. All the patients were middle-aged (above 45). Articles with only male human subjects with full free text were only selected. We excluded articles before 2010, including animal studies, articles in other languages, female subjects, and geriatric populations. The studies with female subjects and the geriatric population were excluded because patients of the geriatric population have a number of associated comorbidities that might affect the outcome of this study. Moreover, HCC occurs more often in males than females; that's why the author wanted the study to focus on male subjects only. Table 2 gives an idea of the MeSH strategy and database.

**Table 2:** MeSH strategy and database MeSH: Medical Subject Headings

MeSH strategy	Database
Diabetes mellitus type 2 OR ("Diabetes Mellitus, Type 2/complications"[Majr] OR "Diabetes Mellitus, Type 2/pathology"[Majr] OR "Diabetes Mellitus, Type 2/physiopathology"[Majr])	PubMed
Nonalcoholic liver disease OR ("Non-alcoholic Fatty Liver Disease/complications"[Majr] OR "Non-alcoholic Fatty Liver Disease/congenital"[Majr] OR "Non-alcoholic Fatty Liver Disease/etiology"[Majr] OR "Non-alcoholic Fatty Liver Disease/pathology"[Majr] OR "Non-alcoholic Fatty Liver Disease/physiopathology"[Majr])	PubMed
Hepatocellular carcinoma OR ("Carcinoma, Hepatocellular/etiology"[Majr] OR "Carcinoma, Hepatocellular/pathology"[Majr] OR "Carcinoma, Hepatocellular/physiopathology"[Majr])	PubMed

Table 3 shows the search strategy and databases used.

 

**Table 3:** Search strategy

Search strategy	Database
Diabetes OR Diabetes mellitus type 2 OR ("Diabetes Mellitus, Type 2/complications"[Majr] OR "Diabetes Mellitus, Type 2/pathology"[Majr] OR "Diabetes Mellitus, Type 2/physiopathology"[Majr] AND Non-alcoholic liver disease OR ("Non-alcoholic Fatty Liver Disease/complications"[Majr] OR "Non-alcoholic Fatty Liver Disease/congenital"[Majr] OR "Non-alcoholic Fatty Liver Disease/etiology"[Majr] OR "Non-alcoholic Fatty Liver Disease/pathology"[Majr] OR "Non-alcoholic Fatty Liver Disease/physiopathology"[Majr] AND Hepatocellular carcinoma OR ("Carcinoma, Hepatocellular/etiology"[Majr] OR "Carcinoma, Hepatocellular/pathology"[Majr] OR "Carcinoma, Hepatocellular/physiopathology"[Majr])	PubMed
Diabetes AND Nonalcoholic fatty liver disease AND Hepatocellular carcinoma	Google Scholar

Result

**Figure 1:**
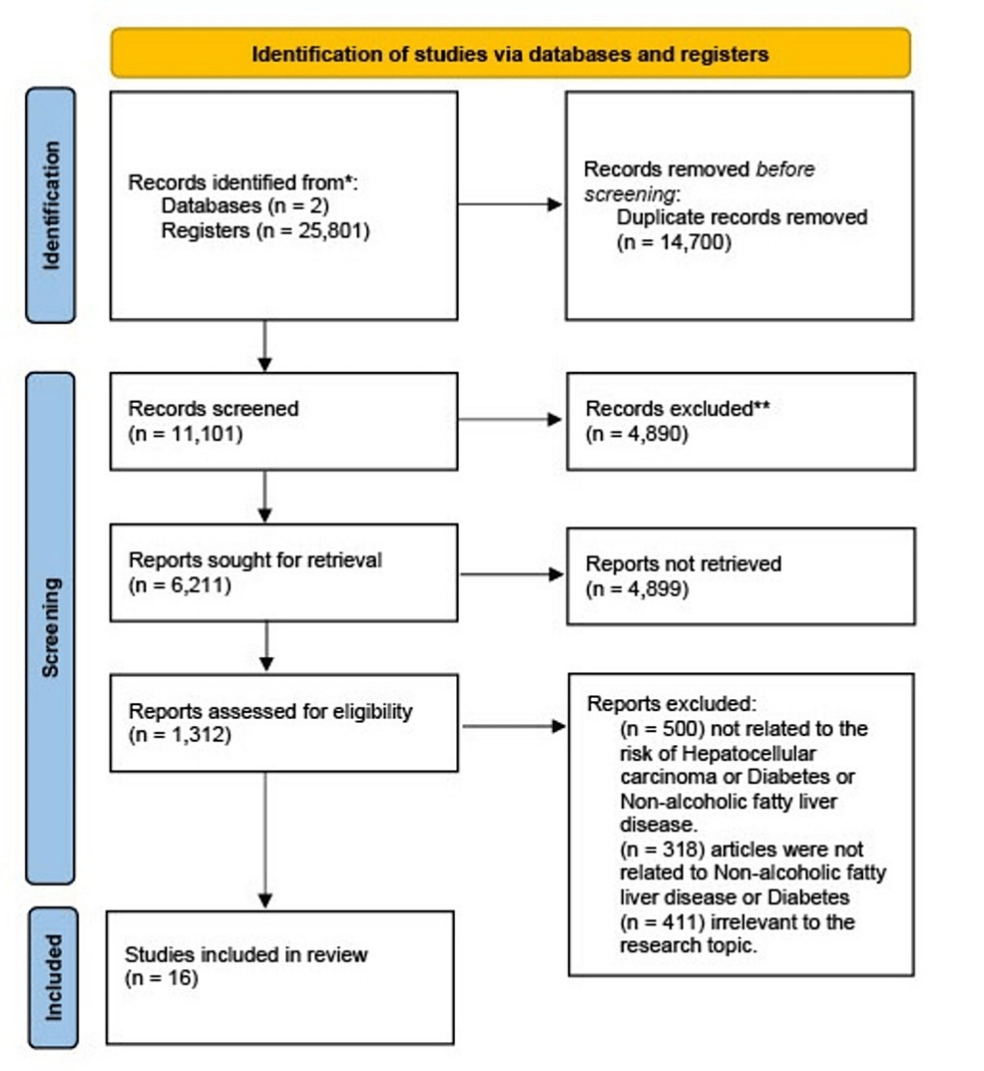
PRISMA flow chart PRISMA: Preferred Reporting Items for Systematic Reviews and Meta-Analyses

Using PubMed and Google Scholar as databases, a total of 9,001 and 16,800 articles were collected using the search strategy and common keywords applying filters such as 2010 to 2021 and free full text in PubMed and 2010-2021 in Google Scholar, respectively. The articles were then screened based on the title and abstract. The articles were filtered on the eligibility criteria and availability of full text. Only 16 articles were available and were examined for quality assessment, and a complete PRISMA flow diagram was created. The studies included in the review underwent quality assessment using tools such as the PRISMA checklist for systematic review and meta-analysis, the Newcastle-Ottawa tool for observational cohort studies, the Joanna Briggs Institute (JBI) checklist tool for case report/series, and the scale for the quality assessment of narrative review articles checklist for literature review. Figure 1 shows the PRISMA flow chart.

Table 4 shows the characteristics of the studies included in the systematic review.

**Table 4:** Characteristics of the studies included NAFLD, nonalcoholic fatty liver disease; HCC, hepatocellular carcinoma; NASH, nonalcoholic steatohepatitis; HTN, hypertension; ALD, alcoholic liver disease; DM, diabetes mellitus

S. no	Author	No of participants	Study type	Study subjects with	Conclusion	Follow-up period
1.	Yang et al. [1]	N = 354	Cohort study	Diabetes and NAFLD	Diabetes is related to an increased risk of HCC in NASH/NAFLD patients	Median of 46 months for diabetics and 47 months for non-diabetics
2.	Than et al. [2]	N = 488	Retrospective study	NAFLD	NAFLD patients with larger tumors are less likely to have curative therapy	Median duration of 1 year
3.	Akuta et al. [3]	N = 402	Retrospective cohort study	NAFLD	HCC most common liver-related event in Japanese patients with histopathologically confirmed NAFLD	Median duration of follow-up was 4.2 years
4.	Ioannou et al. [4]	N = 18,782,281	Cohort study	NAFLD or NASH	Coordinated efforts to identify NAFLD to find the stage of their disease and target the risk progression	Median duration of follow-up was 3.3 years
5.	Hsieh et al. [5]	N = 733	Cohort study	Diabetes mellitus and HTN	DM predicts a better prognosis in advanced HCC treated with sorafenib	_
6.	Amiri Dash Atan et al. [6]	N = 10,897	Systematic review and meta-analysis	NAFLD and diabetes	Overall prevalence of NAFLD among type 2 diabetes mellitus patients is significantly higher	_
7	Wang et al. [7]	N = 9,767	Systematic review and meta-analysis	HCC	Diabetes mellitus is independently associated with both poorer overall survival and poorer disease-free survival in HCC patients	_
8	Wang et al. [9]	_	Systematic review and meta-analysis	HCC and diabetes mellitus	A positive association between diabetes and risk of HCC	Mean follow-up of 8.8 years
9	LI et al. [10]	_	Literature review	DM and HCC	Epidemiological evidence linking DM and HCC	Increase in diabetes mellitus prevalence might increase the incidence of HCC
10	Raff et al. [11]	N = 480	Retrospective cohort	NAFLD/ALD	Diabetes increased the risk of cirrhosis and HCC among ALD and NAFLD	Median follow-up of 3 years
11	Alexander et al. [12]	N = 18,782,281	Cohort study	NAFLD and HCC	Diagnosis of NAFLD/NASH increases the risk of liver outcomes	Mean follow-up of 3.3 years
12	Vernon et al. [13]	_	Systematic review	NAFLD/NASH	NAFLD prevalence and impact continue to increase, making NASH potentially the most common cause of advanced liver disease	_
13	Younossi et al. [14]	N = 19,916	Cohort study	HCC	Increased risk of HCC in patients with NAFLD	_
14	Ertle et al. [15]	N = 162	Cohort study	NAFLD	With the rapid increase in the incidence of MS, NAFLD, and associated comorbidities, it is clear that clinicians are well-educated and aware	_
15.	Wong et al. [16]	N = 296,707	Retrospective cohort study	NAFLD	Risk of HCC was higher in patients with NAFLD than in the general clinical population	Mean follow-up of 9.0 and 8.67 years
16.	Limaiem et al. [17]	N = 64	Retrospective study (case series)	HCC	HCC is associated with a high rate of mortality	Mean follow-up of 26 months

Discussion

In this study, we considered diabetes as a key risk factor for HCC in patients with NAFLD. Many risk determinants of HCC were already discussed such as the etiology of cirrhosis, age, gender, race, BMI, diabetes, and HCV [4]. Among the discussed risk factors, diabetes has a significant association with HCC [9].

Diabetes and HCC

In a study, Amiri Dash Atan et al. concluded that NAFLD is common among the type 2 diabetes mellitus population. In a study conducted with 354 patients, it was concluded that out of 354 patients, 253 individuals (71%) had diabetes, of which 67% of patients died of end-stage liver diseases including HCC. Individuals with higher age profiles, low albumin, and diabetes are all related to an increased risk of HCC [1]. The association between diabetes and HCC, which pose the sixth most common cancer worldwide, was reported by Lawson et al. in 1986, and about 11% of cancer-associated deaths are from HCC. The progression of HCC due to insulin and insulin-like "growth factor 1" (IGF-1) exhibits its proliferative effects on cells, oncogenic effects of hyperglycemia, and obesity. A study by Adami et al. in Sweden expressed the risk of pancreatic and hepatocellular cancers in relation to diabetes mellitus [10]. Another study systematically reviewed epidemiologic investigation on diabetes mellitus and HCC in Japanese populations which showed a 9 out of 10 odds ratio in case-control studies and 17 out of 24 relative risks in cohort studies between diabetes mellitus and HCC, specifying that there is an increased risk of HCC in diabetes mellitus patients. Table 5 shows the increase in the cumulative probability of HCC in both patients with diabetes and those who do not have diabetes.

**Table 5:** Cumulative probability of HCC in diabetic and non-diabetic patients over the years

No of years	Diabetic	Non-diabetic
1	0.05	0.03
2	0.06	0.03
3	0.1	0.06
4	0.1	0.06
5	0.13	0.07
6	0.13	0.07
7	0.17	0.1
8	0.2	0.1

It is evident that there is an increased risk of HCC in patients who have developed diabetes [11].

Association between NAFLD and HCC

**Figure 2:**
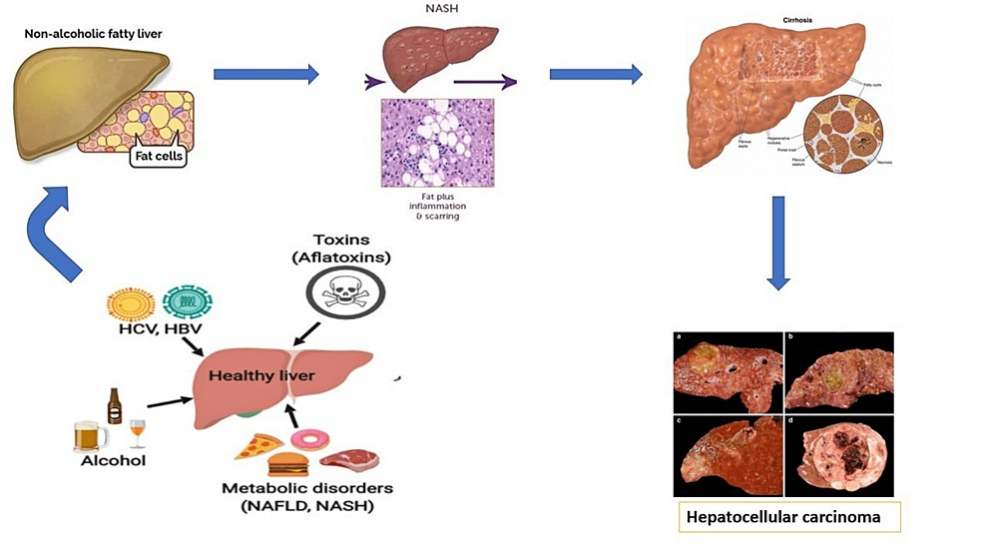
Stages of liver cancer development from NAFLD to end-stage HCC Originally done by the first author NAFLD, nonalcoholic fatty liver disease; NASH, nonalcoholic steatohepatitis; HCV, hepatitis C virus; HBV, hepatitis B virus

In a study by Alexander et al., 531,452 persons were taken as patients with NAFLD and 43,385,495 persons as controls. Among NAFLD/nonalcoholic steatohepatitis (NASH) patients, the incidence of cirrhosis was 0.76 per 1000 person-years with a 95% confidence interval (CI) of 0.46 to 2.32, and the incidence of HCC diagnosis was 0.3 per 1000 person-years of all the patients with NAFLD/NASH with 95% CI of 0.26 to 0.60. There was a greater risk of new cases of HCC when compared with controls [12]. In people who have preexisting metabolic disorders like obesity and type 2 diabetes mellitus, NAFLD is found to increase. Frith et al. conducted a study of 351 patients with NAFLD (biopsy proven) who were categorized as elderly (60 above), middle-aged (50 to 60), and young (below 50) in which relationships between the prevalence of NAFLD with fibrosis and age is shown. Older patients have more risk factors for NAFLD such as hypertension, obesity, diabetes, and hyperlipidemia [13]. Nowadays, studies suggest that these risk factors are absent in a major proportion (20-40%) of HCC of which not all of them have NAFLD as an underlying etiology. A study result indicated that the rate of HCC reported to the Surveillance, Epidemiology, and End Results Program has increased. This increase is not only associated with an increase in the prevalence of NAFLD but also with an increase in the awareness of HCC. NAFLD and HCC patients are elderly at the time of diagnosis with a survival rate of fewer than 5 months; moreover, in NAFLD-associated HCC, 62% of patients died within a year because of poor prognosis [14]. By taking most of the study and the results into consideration and follow-up with NAFLD patients from various studies into reference, it is clear that metabolic disorders like obesity and diabetes play a vital role in developing liver complications in NAFLD patients. In our case, diabetes plays a pivotal role in NAFLD patients since it may cause disease progression from NAFLD to more evolved liver complications such as NASH and even to advanced fibrosis with HCC end-stage complications. Figure 2 shows the stages of the progression of HCC.

Pathophysiology

**Figure 3:**
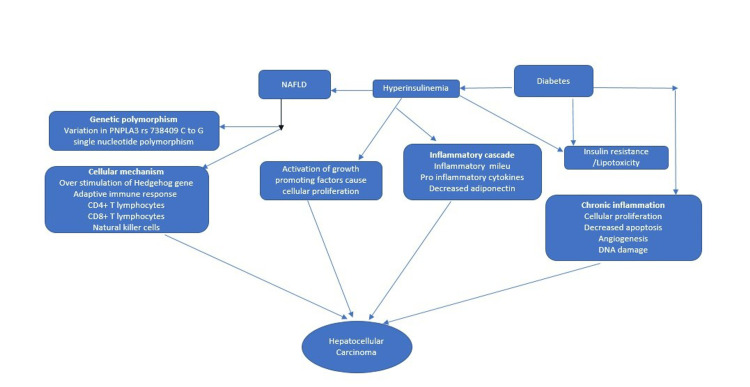
Correlation between NAFLD, diabetes, and HCC Done by the first author NAFLD: nonalcoholic fatty liver disease [15-17]

It is unclear exactly how diabetes, NAFLD, and HCC are linked, but recently, we have a better knowledge of the pathophysiology of HCC [1]. Carcinogenesis in the liver is promoted by a variety of mechanisms such as the production of proinflammatory cytokines in inflammatory cascades and ROS that can cause genetic instability, increase the proliferation of cells, and prevent apoptosis in liver hepatocytes [15-16]. Figure 3 describes the development of HCC in relation to NAFLD and diabetes.

Metabolic disorders like diabetes can cause insulin resistance (peripheral/hepatic), lipotoxicity, chronic inflammation (low grade), and oxidative which all lead to increased cellular growth/proliferation, decreased apoptosis, increased angiogenesis, and increased DNA damage to the hepatocytes, resulting in the development of HCC in the setting of diabetes.

In NAFLD, several mechanisms were suggested:

1. Cellular mechanism causes over-stimulation of the hedgehog signaling and may lead to dysregulated cellular repair which causes impaired replacement of damaged hepatocytes with liver progenitor cells leading to malignant transformation of hepatic progenitor cells.

2. In genetic polymorphism variation in PNPLA3 rs738409 C to G single nucleotide polymorphism.

3. In the setting of hyperinsulinemia activation of growth-promoting factors such as IGF-1 and M6P/IGF2R (tumor suppressor gene), IRS1 causes cellular proliferation, and in inflammatory cascade inflammatory milieus like free fatty acids, pro-inflammatory cytokines, ROS, JNK1, and decreased adiponectin, an anti-inflammatory polypeptide can cause further development of HCC [15-17].

Tumor suppressor genes

Phosphatase and tensin homolog and p53 tumor suppressor genes play a vital role in the incidence of steatosis and thus cause liver damage. Inactivation of tumor suppressor genes can lead to HCC; poorly controlled NF-jB, IL-6, and TAK1 signaling can also cause HCC [15].

Hedgehog signaling

Hedgehog signaling plays an important role in replacing damaged liver cells with hepatic progenitor cells, helping in cellular repair. With the change in the normal hedgehog signaling pathway, there is a decline in liver progenitor cells which decreases cellular repair and leads to the uncontrolled proliferation of aberrant hepatocytes. In NAFLD, excessive hedgehog activity may increase the lifespan of progenitor cells and cellular differentiation which may cause further disease progression [15-16].

NAFLD and HCC

Several mechanisms were suggested for the development of HCC such as chronic inflammation in the condition of hyperinsulinemia or metabolic syndrome, hepatic progenitor cells and adaptive immune responses, and genetic polymorphism patatin like PNPLA3 [16].

Genetic and other risks

In the NAFLD-HCC pathogenesis, there is variation in PNPLA3. About 9,229 genetic variants of PNPLA3 showed that homozygous carriers of the I148M variant protein of PNPLA3 had twice the greater hepatic fat content than non-carriers, and I148M was more common in the group who were at the highest risk of NAFLD. In a study done by Liu et al, it was concluded that PNPLA3 rs738409 C_G single nucleotide polymorphism encoded the I148M variant protein and demonstrated a gene dosage effect with an increased number of homozygous G alleles. It was found that a higher incidence of NAFLD-associated HCC has an odds ratio of 12:19. It is high when compared to the general population of the United Kingdom, and this PNPLA3 rs738409 C_G polymorphism has been linked with a higher risk of NAFLD-related HCC, independent of cirrhosis [16]. HCV, heavy alcohol drinking, and hepatitis B virus infection are significant risk factors for HCC. HCC occurs more often in older than in young, affecting males more than females at a ratio of 2:1 to 4:1 [17].

Limitations

There are some limitations to this study. Articles from 2010 to 2021 were only selected; due to this, there is a chance that we may have overlooked articles that were published before 2010. We only included middle-aged men (above 45), and only articles written in the English language were taken. We did not consider other genders and age group populations in this study.

## Conclusions

Even though there are many risk determinants for HCC, diabetes is one of the noteworthy risk determinants. We understand diabetes in obese patients is known to cause major complications that affect vital organs such as the kidney, heart, brain, and liver. It is important not only for the elderly but also people of all age groups to monitor their hemoglobin A1C levels to prevent the complications of diabetes. Lifestyle modification can reduce the risk of developing complications like HCC. Physical activity and diet restriction itself can have a major impact on health and decrease comorbidities like diabetes and NAFLD.
